# Successful Extended Allopurinol Desensitization in an Elderly Patient With Chronic Kidney Disease and Febuxostat Intolerance

**DOI:** 10.7759/cureus.105710

**Published:** 2026-03-23

**Authors:** Rommel Ramesh, Vivek Nagaraja, Alexei Gonzalez-Estrada

**Affiliations:** 1 Department of Allergy, Asthma and Clinical Immunology, Mayo Clinic, Phoenix, USA; 2 Department of Rheumatology, Mayo Clinic, Phoenix, USA

**Keywords:** allopurinol, desensitization, drug hypersensitivity reaction, febuxostat intolerance, gout

## Abstract

Effective management of gout in patients with chronic kidney disease (CKD) is frequently complicated by medication intolerance. Although allopurinol remains the preferred first-line urate-lowering therapy, cutaneous hypersensitivity reactions often necessitate drug discontinuation. Febuxostat is commonly used as an alternative, but intolerance or adverse effects may limit its use in certain patients. We describe a therapeutic solution for a patient who failed both standard agents.

An 82-year-old woman with CKD and tophaceous gout presented with a benign maculopapular eruption following allopurinol initiation. After discontinuation of the drug, febuxostat was initiated, but this trial was aborted due to idiosyncratic symptomatic hypotension (94/64 mmHg). With no other oral options available, the patient underwent a conservative, 28-day oral allopurinol desensitization protocol under corticosteroid coverage. She successfully completed the desensitization and was titrated to a maintenance dose of 150 mg daily, achieving target serum uric acid levels without recurrence of skin manifestations.

This case illustrates that a prolonged "start low, go slow" desensitization protocol can be safely employed in elderly patients with CKD who have experienced benign cutaneous reactions to allopurinol. This approach is particularly valuable when alternative agents like febuxostat are contraindicated or poorly tolerated, ensuring that patients are not left without effective treatment for progressive gout.

## Introduction

Gout is a crystal-induced articular disease with heterogeneous presentations and an increasing global prevalence. Allopurinol, a xanthine oxidase inhibitor, is a first-line urate-lowering pharmacotherapy. While allopurinol is generally well-tolerated, approximately 2% of patients experience cutaneous hypersensitivity reactions, most commonly mild maculopapular exanthema [1]. Severe cutaneous adverse reactions including Stevens-Johnson syndrome (SJS), toxic epidermal necrolysis (TEN), and drug reaction with eosinophilia and systemic symptoms (DRESS) can occur in approximately 0.4% of cases and carry significant mortality [2].

The pathophysiology of these reactions is often attributed to oxypurinol, the major active metabolite of allopurinol. Oxypurinol accumulation is particularly problematic in patients with renal impairment due to its prolonged half-life, placing this cohort at elevated risk [3]. This poses a challenge in the management of patients with gout who may have limited alternative options for urate-lowering therapy. Extended desensitization protocols offer a vital therapeutic pathway for patients with delayed benign hypersensitivity reactions when alternative therapies fail. We present a case of successful extended allopurinol desensitization in a patient for whom febuxostat was not a viable option due to documented drug-induced hypotension.

## Case presentation

An 82-year-old female with a past medical history of chronic kidney disease (CKD), coronary artery disease, hypertension, osteoporosis, fatty liver, and primary hyperparathyroidism (status post partial parathyroidectomy) presented for the management of recalcitrant tophaceous gout. She reported past alcohol consumption limited to 1-2 glasses of wine monthly. Radiographic imaging of the first metatarsophalangeal joint revealed severe osteoarthritis with osteophytic ridging and hallux rigidus; notably, no chondrocalcinosis was identified. Her baseline serum uric acid (sUA) levels ranged from 7.5 to 9.0 mg/dL (reference 3.5-7.2 mg/dL), and triglycerides were 129 mg/dL (reference <150 mg/dL).

The patient’s index reaction to allopurinol manifested two weeks after allopurinol initiation as a pruritic, maculopapular exanthema distributed across the thorax and upper extremities. The reaction was classified as a benign delayed hypersensitivity reaction; there was no mucosal involvement, fever, or lymphadenopathy. HLA-B*58:01 testing, which is associated with increased risk of severe allopurinol hypersensitivity, was not performed. Although she was taking losartan 25 mg daily, a medication with mild uricosuric properties, her sUA levels remained above target. Fenofibrate was not indicated, given her normal triglyceride levels.

Withdrawal of allopurinol led to complete resolution of the rash within one week, without the need for supportive care. Subsequent management with febuxostat was attempted; however, this trial was complicated by symptomatic hypotension with blood pressure measured at 94/64 mmHg. She reported immediate improvement after discontinuation of febuxostat, and the drug was not rechallenged. Because other uricosuric agents were contraindicated due to her CKD (serum creatinine 1.1 mg/dL; estimated glomerular filtration rate (eGFR) 50 mL/min/1.73 m²), the patient was identified as a candidate for extended allopurinol desensitization.

An extended, 28-day oral allopurinol desensitization protocol (Table 1) was designed to mitigate the risk of recurrent hypersensitivity based on 2010 drug allergy practice parameters [4]. The patient was maintained on concomitant low-dose systemic corticosteroids to suppress potential allergic responses and prevent acute gout flares. She successfully completed the protocol, reaching the initial target dose of 100 mg daily without cutaneous recurrence.

**Table 1:** Allopurinol Extended Desensitization Protocol ^1^SuspendRx™ Anhydrous (oil-based, sugar-free) diluent

Steps	Daily dose (mg)	Concentration^1^	Amount	Days
Step 1	0.05	0.1 mg/mL	0.5 mL	1-3
Step 2	0.1	0.1 mg/mL	1 mL	4-6
Step 3	0.2	0.1 mg/mL	2 mL	7-9
Step 4	0.5	0.1 mg/mL	5 mL	10-12
Step 5	1	1 mg/mL	1 mL	13-15
Step 6	5	1 mg/mL	5 mL	16-18
Step 7	10	1 mg/mL	10 mL	19-21
Step 8	25	100 mg/ tablet	1/4 tablet daily	22-24
Step 9	50	100 mg/ tablet	1/2 tablet daily	25-27
Step 10	100	100 mg/ tablet	1 tablet daily	≥28

At the three-month follow-up, the allopurinol dose was up-titrated to 150 mg daily to achieve target sUA goals. The patient remained free of any maculopapular exanthema or other hypersensitivity reactions.

## Discussion

Allopurinol remains the cornerstone of urate-lowering therapy; however, its utility is frequently limited by cutaneous adverse reactions. This case highlights a complex therapeutic dilemma: an elderly patient with CKD who experienced a delayed hypersensitivity reaction to allopurinol and subsequently failed febuxostat due to hypotension. Desensitization represents a viable pathway to prevent the progression of tophaceous gout in this specific phenotype.

Allopurinol hypersensitivity arises from the intersection of pharmacokinetics and genetics. First, in patients with CKD, the active metabolite oxypurinol accumulates due to reduced renal clearance, creating a high antigenic load [3]. Second, in susceptible individuals carrying the HLA-B*58:01 allele, this excess oxypurinol binds to the HLA molecule, altering its structure [5]. This "altered self" complex mimics a foreign antigen, tricking CD8+ cytotoxic T-cells into launching an immune attack against cutaneous tissue, resulting in the characteristic exanthema [5]. Given this well-established mechanism, current pharmacogenetic guidelines strongly advocate for HLA-B*58:01 screening prior to allopurinol initiation in specific high-risk populations to mitigate the risk of severe cutaneous adverse reactions [6]. While baseline screening was not indicated for our patient due to her Caucasian background, her benign index presentation made her an appropriate candidate for subsequent desensitization.

A critical aspect of this case was the selection of a prolonged, conservative 28-day desensitization protocol. Given the patient's age and renal comorbidities, a rapid introduction carries a higher risk of systemic toxicity. By utilizing a "start low, go slow" approach, we allowed for gradual enzymatic adaptation and the potential induction of immune tolerance. This aligns with established data suggesting that slower titration, particularly in CKD patients, significantly reduces the risk of recurrent hypersensitivity [7].

Current American College of Rheumatology guidelines recommend switching to febuxostat as the primary alternative for patients with allopurinol hypersensitivity [8]. However, our patient experienced symptomatic hypotension upon initiation. While febuxostat has been scrutinized for cardiovascular safety, with the CARES trial noting increased cardiovascular mortality [9] and the subsequent FAST trial demonstrating non-inferiority to allopurinol [10], acute symptomatic hypotension is an infrequently reported adverse event. This rarity makes the clinical presentation unique and limits the guidance available for clinicians. Consequently, this failure underscores the necessity of maintaining allopurinol desensitization as a clinical competency, as reliance on second-line pharmacotherapy is not always feasible.

## Conclusions

In elderly patients with CKD and benign allopurinol hypersensitivity, a 28-day “start low, go slow” desensitization protocol is a safe and effective strategy. It allows for resumption of first-line urate-lowering therapy, especially when other therapeutic options have been exhausted, ensuring effective long-term management of tophaceous gout.
